# National Variation in Implementation of Sentinel Lymph Node Biopsy for Clinically Node-Positive Patients Undergoing Neoadjuvant Therapy

**DOI:** 10.1245/s10434-025-17293-x

**Published:** 2025-04-18

**Authors:** Crystal D. Taylor, Ton Wang, Brandy R. Sinco, Melissa Pilewskie, Tasha M. Hughes, Lesly A. Dossett

**Affiliations:** 1 Department of Surgery, Michigan Medicine, Ann Arbor, MI USA; 2 Center for Health Outcomes and Policy, Michigan Medicine, Ann Arbor, MI USA; 3 Division of Surgical Oncology, Institute for Healthcare Policy and Innovation, University of Michigan, Ann Arbor, MI USA; 4 National Clinician Scholars Program, University of Michigan, Ann Arbor, MI USA; 5 Department of Surgery, Duke University, Durham, NC USA

## Abstract

**Background:**

Sentinel lymph node biopsy (SLNB) is feasible in women with clinically node-positive breast cancer following neoadjuvant chemotherapy and a nodal pathologic complete response. Acceptable false negative rates are achieved through technical considerations such as removing three or more sentinel lymph nodes (SLNs); however, the variation that exists in adherence to this technique is unclear.

**Objective:**

This study aimed to evaluate recent trends in axillary surgery in women with cN1-cN2 disease who received neoadjuvant chemotherapy, adherence to removing three or more SLNs, and variation in SLN yield.

**Methods:**

We performed a cohort study using the National Cancer Database of women aged ≥18 years with cN1-cN2 disease who received neoadjuvant chemotherapy, including those without a pathologic complete response, from 2012 to 2020. Trends in axillary surgery and lymph node yield obtained during SLNB were evaluated.

**Results:**

The cohort included 67,365 women (median age 54 years). The number of patients receiving SLNB alone increased from 14 to 39%; SLNB with completion axillary lymph node dissection (ALND) increased from 17 to 30%; and ALND alone decreased from 69 to 27%. The rates of obtaining three or more SLNs during SLNB remained the same over time at 66%, while facility-level variation in obtaining three or more nodes ranged from 40 to 86%.

**Conclusions:**

There has been de-escalation of axillary surgery with fewer patients undergoing ALND; however, overall there has been no significant change in the rates of obtaining three or more lymph nodes during SLNB following neoadjuvant chemotherapy, with significant facility-level variation observed.

**Supplementary Information:**

The online version contains supplementary material available at 10.1245/s10434-025-17293-x.

Axillary surgery provides prognostic information and informs adjuvant therapy decisions in patients with invasive breast cancer. Previously, the standard of care for women with clinically node-positive (cN+) breast cancer was to receive axillary lymph node dissection (ALND) for appropriate nodal staging irrespective of response to neoadjuvant chemotherapy. Results of multiple prospective trials have demonstrated that sentinel lymph node biopsy (SLNB) is feasible in women with cN1 disease following neoadjuvant chemotherapy, with an acceptable false negative rate of < 10%,^1–4^ achievable through specific technical modifications, including dual tracer mapping, identification of the clipped biopsy-proven positive node, and removing three or more sentinel lymph nodes (SLNs).^1–6^ Following demonstration of feasibility, multiple retrospective reports have shown that SLNB is an appropriate alternative to ALND among patients who achieve a nodal pathologic complete response (pCR).^7–11^ Omission of SLNB among patients with cN1 disease who achieve a nodal pCR following neoadjuvant chemotherapy can spare over 40% of eligible node-positive patients an ALND and the associated morbidities, most notably lymphedema.^7,12,13^

Current trends in axillary surgery now demonstrate a shift in practice, with increasing rates of SLNB performed in cN+ patients with breast cancer undergoing neoadjuvant chemotherapy. Since the results of the American College of Surgeons Oncology Group (ACOSOG) Z1071 and SENTINA trials were published in 2013, the national rates of ALND have decreased substantially in the upfront surgery setting.^10,14–16^ Despite the implementation of SLNB into the treatment algorithm for cN+ breast cancer following neoadjuvant chemotherapy, it is unclear to what extent there may be variation in the technical performance of the procedure. We previously demonstrated significant variation in nodal yield for SLNB in clinically node-negative patients.^17^ Similar variation in cN+ patients could increase the false negative rate of SLNB in these patients, impacting adjuvant therapy recommendations and possibly leading to worse outcomes.

Changes in clinical practice and technical guidelines for SLNB post-neoadjuvant chemotherapy have yet to be clearly established and broadly reported since these studies were published. Organizations such as the National Comprehensive Cancer Network (NCCN) have supported the use of clipping and retrieving the biopsied node, use of dual tracer, and obtaining three or more SLNs to minimize false negative rates.^18^ We sought to review the specific uptake of removing three or more SLNs to ascertain the clinical implementation of performing less aggressive axillary surgery post-neoadjuvant chemotherapy. To evaluate the implementation of this trial data into practice, we evaluated trends in axillary surgery in women with cN1-cN2 disease who received neoadjuvant chemotherapy. We then investigated adherence to recommendations for removing three or more SLNs and variation in SLN yield between facilities.

## Methods

We conducted a cohort study using the National Cancer Database (NCDB) to determine trends in axillary surgery and lymph node yield during SLNB following neoadjuvant chemotherapy in cN+ breast cancer. This study was deemed exempt by the University of Michigan Institutional Review Board and follows the Strengthening the Reporting of Observational Studies in Epidemiology (STROBE) guidelines for cohort studies.^19^

### Data Source

The NCDB is a national clinical oncology database containing hospital registry data from more than 1500 Commission on Cancer-accredited facilities.^20^ It is the largest clinical cancer registry in the world, containing over 34 million records, and captures approximately 70% of new cancer diagnoses.^21,22^ All data were de-identified and Health Insurance Portability and Accountability Act compliant.

### Study Population

The cohort included women aged ≥18 years with cN1-cN2 breast cancer who received neoadjuvant chemotherapy, including women without a pCR, from 2012 to 2020. Women with stage IV breast cancer and inflammatory breast cancer, as well as those who did not receive neoadjuvant chemotherapy, had cT0 disease, or received treatment outside of their primary facility were excluded. Missing observations based on our selection criteria were omitted from this study. Only facilities that performed an average of 10 procedures annually were included in order to obtain a reliable estimate of facility performance. We conducted further analyses based on volume and facility type. Hospital volume was based on the average annual breast cancer case count and was categorized as low (10–99 cases), medium (100–199 cases), or high (≥ 200 cases). These cut-offs were chosen based on previously published ranges to ensure adequate facility- and patient-level data across groups for multilevel analysis.^23,24^

### Outcome Variables

Trends in axillary surgery (SLNB alone, SLNB with completion ALND, or ALND alone) following neoadjuvant chemotherapy were evaluated from 2012 to 2020. Each axillary surgery was classified using the RXSummScopeRegLN2012 variable in the NCDB. To evaluate lymph node yield during SLNB, the SLNEXAM variable was used to evaluate patients undergoing SLNB and quantify the number of lymph nodes examined. As this dedicated variable to the SLN examination was unavailable before 2018 in the NCDB, we opted not to use lymph node yield data from 2012 to 2017 where only a surrogate variable was available. Additionally, evaluation of trends in axillary surgery for cN+ patients with a pCR only, categorized as pathologic N0 disease, was then performed to examine if these trends remained. To quantify the number of lymph nodes removed during ALND and SLNB with completion ALND (ALND performed in the same operation or a subsequent operation), the RegionalNodesExamined variable was used. While the NCDB includes lymph node values of up to 90 or more, we limited the number of possible lymph nodes examined to 40 (values over 40 were considered inaccurate and were excluded from our analysis).

### Statistical Analysis

Reliability-adjusted estimates for the rates of SLNB, ALND, and SLNB with completion ALND were generated from logistic regression models with a random intercept for each facility, also known as generalized linear mixed models (GLMMs) with a logit link. All models used to compare surgical procedure rates based on facility characteristics had binary outcomes for the surgical procedure, random intercepts for hospitals, and covariates of (time in years from 2012) facility type, facility location, facility volume category, and an interaction between (time in years from 2012) and the facility characteristic that was being assessed for differences. For example, the model used to compare facility types contained an interaction between (facility type) × (time in years from 2012). The model used to compare facility locations had an interaction *n* between (facility location) × (time in years from 2012), and the model to compare volume categories contained the interaction, (volume category) × (time in years from 2012).

Similarly, the reliability-adjusted estimates for nodal positivity were also computed from GLMMs for logistic regression with random intercepts for hospitals. A GLMM with a hospital-level random intercept accounts for clustering within hospitals and is equivalent to an empirical Bayesian estimate.^25^ The time series estimates were computed from GLMMs with a cubic polynomial for ‘years since 2012’ and random intercepts for each hospital. Multiple comparisons using Monte Carlo simulation were applied to evaluate for differences in trends in the rates of obtaining three or more SLNs.^26^ A *p*-value of < 0.05 was considered significant and all *p*-values were two-sided. All statistical analyses were performed using SAS statistical software, version 9.4 (SAS Institute Inc., Cary, NC, USA).

## Results

### Study Population

**Fig. 1:**
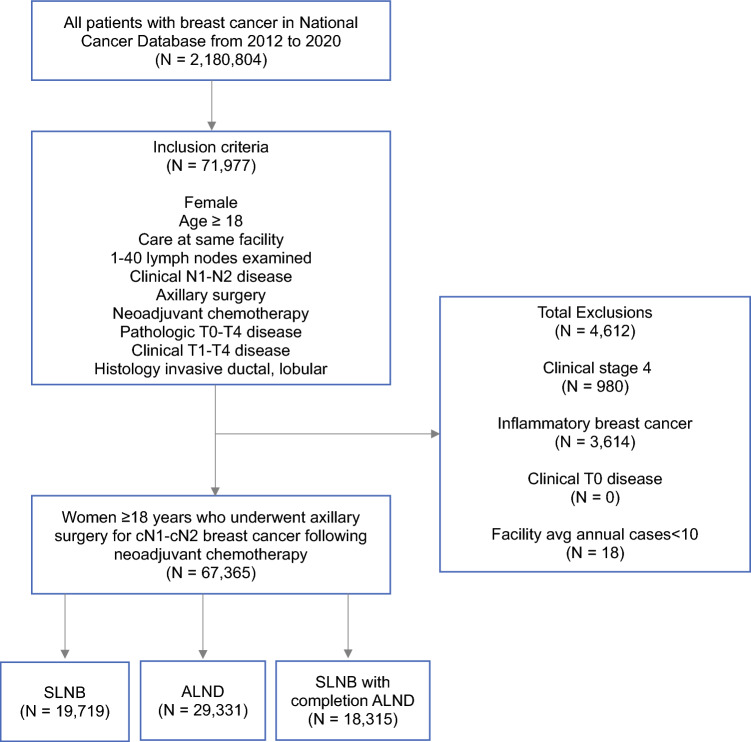
Study consort diagram. *SLNB* sentinel lymph node biopsy, *ALND* axillary lymph node dissection

After inclusion and exclusion criteria were applied, 67,365 women with cN1-cN2 disease who received neoadjuvant chemotherapy from 2012 to 2020 were identified (Fig. 1). Median age was 54 years (interquartile range [IQR] 45–64). Overall, 90% (*n *= 60,740) of women had cN1 disease and 10% (*n *= 6625) had cN2 disease. With respect to axillary surgery performed, 29% (*n *= 19,719) of women underwent SLNB, 27% (*n *= 18,315) underwent SLNB with completion ALND, and 44% (*n *= 29,331) underwent ALND following neoadjuvant chemotherapy (Table 1). A total of 1306 facilities were included in the analysis. Patient distribution according to facility characteristics are reported in Table 1. In brief, 32% (*n *= 21,708) of patients were treated at a comprehensive cancer center, 30% (*n *= 19,913) were treated at an academic center, 20% (*n *= 13,349) were treated at an integrated network, and 5% (*n *= 3532) were treated at a community cancer center. Most patients were treated at a high-volume facility (69%, *n *= 46,635).

**Table 1:** Characteristics of women with clinically node-positive breast cancer undergoing axillary surgery following neoadjuvant chemotherapy

Characteristic	
Age, years [mean]	54.5 (12.7)
Age, years [median (IQR)]	54 (45–64)
*Race*	
White	49,225 (73.1)
Black	12,784 (19.0)
Asian	3174 (4.7)
Native American	393 (0.6)
Other race	1745 (2.6)
Hispanic ethnicity	6511 (9.7)
Unknown	1177 (1.7)
*Tumor characteristics*	
*Clinical T stage*	
T1	11,467 (17.0)
T2	34,426 (51.1)
T3	15,498 (23.0)
T4	5974 (8.9)
*Clinical N stage*	
N1	60,740 (90.2)
N2	6625 (9.8)
*Pathologic T stage*	
T0	15,270 (22.7)
T1	25,865 (38.4)
T2	17,498 (26.0)
T3	6726 (10.0)
T4	2006 (3.0)
*Pathologic N stage*	
N0	23,474 (34.8)
N1	27,301 (40.5)
N2	11,698 (17.4)
N3	4615 (6.9)
Other	277 (0.4)
*Tumor histology*	
Invasive lobular carcinoma	58,209 (86.4)
Invasive ductal carcinoma	4825 (7.2)
Both ductal and lobular	2391 (3.5)
Other/unknown	1940 (2.9)
*Tumor receptor status*	
HR−/HER2−	17,381 (25.8)
HR+/HER2−	36,690 (54.5)
HR−/HER2+	7639 (11.3)
HR+/HER2+	4254 (6.3)
Unknown	1401 (2.1)
*Surgery performed*	
Lumpectomy	44,011 (65.3)
Mastectomy	23,324 (34.6)
Unknown	30 (0.1%)
*Axillary surgery*	
SLNB	19,719 (29.3)
SLNB with completion ALND	18,315 (27.2)
ALND	29,331 (43.5)
*Facility type*	
Community cancer	3532 (5.2)
Comprehensive center	21,708 (32.2)
Academic center	19,913 (29.6)
Integrated network	13,349 (19.8)
Unknown	8863 (13.2)
*Annual case volume*	
Low (<100)	6541 (9.7)
Medium (100–199)	14,189 (21.1)
High (≥200)	46,635 (69.2)

Data are expressed as *n* (%) unless otherwise specified

*IQR* interquartile range, *HR* hormone receptor, *HER2* human epidermal growth factor receptor 2, *SLNB* sentinel lymph node biopsy, *ALND* axillary lymph node dissection

### Trends in Axillary Surgery

**Fig. 2:**
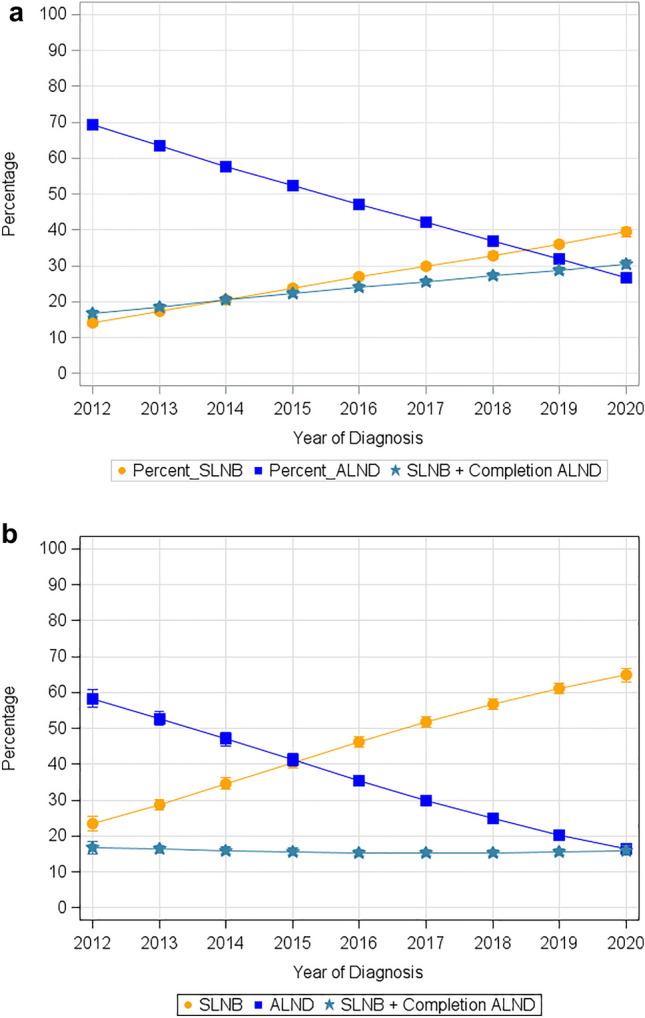
Trends in axillary surgery over time in patients with cN1-cN2 disease following (**a**) neoadjuvant chemotherapy from 2012 to 2020, and (**b**) neoadjuvant chemotherapy with a pathologic complete response from 2012 to 2020. *SLNB* sentinel lymph node biopsy, *ALND* axillary lymph node dissection

Trends in axillary surgery for patients with cN1 and cN2 disease following neoadjuvant therapy are noted in Table 2 and Fig. 2a. From 2012 to 2020, the number of patients receiving SLNB increased from 14 to 39%, SLNB with completion ALND increased from 17 to 30%, and ALND decreased from 69 to 27%. Similar trends were seen in patients with pathologic N0 disease considered to have a pCR. From 2012 to 2020, the number of patients with a pCR receiving SLNB increased from 23 to 65%, SLNB with completion ALND decreased from 17 to 16%, and ALND decreased from 58 to 16% (Table 3 and Fig. 2b). Across facility type and volume, there were no significant differences in the rate of implementation of SLNB and the de-escalation of ALND following neoadjuvant chemotherapy.

**Table 2:** Trends in axillary surgery in clinically node-positive breast cancer following neoadjuvant chemotherapy

Year	SLNB	ALND	SLNB with completion ALND
2012	14.0 (13.1, 14.9)	69.3 (67.9, 70.7)	16.7 (15.7, 17.7)
2013	17.3 (16.6, 18.1)	63.4 (62.2, 64.6)	18.6 (17.9, 19.4)
2014	20.7 (19.9, 21.5)	57.8 (56.5, 59.0)	20.4 (19.6, 21.3)
2015	23.9 (23.0, 24.7)	52.4 (51.1, 53.6)	22.2 (21.4, 23.0)
2016	26.9 (26.1, 27.8)	47.1 (46.0, 48.3)	23.9 (23.1, 24.7)
2017	29.9 (29.0, 30.8)	42.0 (40.9, 43.2)	25.5 (24.7, 26.4)
2018	32.9 (31.9, 33.8)	36.9 (35.8, 38.1)	27.1 (26.2, 28.0)
2019	36.0 (35.0, 37.0)	31.8 (30.8, 32.9)	28.7 (27.8, 29.6)
2020	39.4 (38.2, 40.7)	26.6 (25.4, 27.8)	30.4 (29.2, 31.6)

Data are expressed as percentages

*SLNB* sentinel lymph node biopsy, *ALND* axillary lymph node dissection

**Table 3:** Trends in axillary surgery in clinically node-positive breast cancer following neoadjuvant chemotherapy with a pathologic complete response

Year	SLNB	ALND	SLNB with completion ALND
2012	23.4 (21.5, 25.4)	58.3 (55.8, 60.8)	16.6 (15.0, 18.4)
2013	28.8 (27.3, 30.3)	52.8 (50.9, 54.6)	16.3 (15.2, 17.4)
2014	34.5 (32.9, 36.2)	47.0 (45.1, 48.9)	15.9 (14.9, 17.1)
2015	40.5 (38.8, 42.1)	41.2 (39.5, 42.9)	15.6 (14.6, 16.6)
2016	46.3 (44.7, 47.8)	35.4 (33.9, 37.0)	15.3 (14.4, 16.3)
2017	51.7 (50.2, 53.3)	30.0 (28.5, 31.5)	15.2 (14.3, 16.1)
2018	56.7 (55.2, 58.3)	24.9 (23.6, 26.3)	15.2 (14.3, 16.2)
2019	61.2 (59.7, 62.6)	20.3 (19.2, 21.5)	15.5 (14.5, 16.4)
2020	64.9 (63.0, 66.8)	16.3 (15.0, 17.7)	16.0 (14.7, 17.4)

Data are expressed as percentages

*SLNB* sentinel lymph node biopsy, *ALND* axillary lymph node dissection

### Sentinel Lymph Node Biopsy Lymph Node Yield

**Fig. 3:**
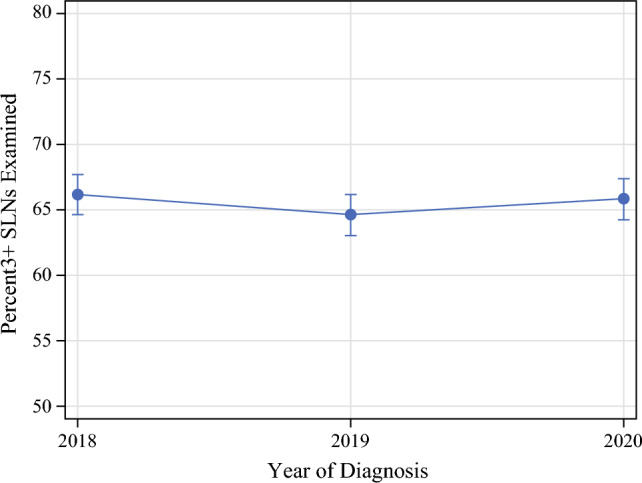
Trends in obtaining three or more SLNs during sentinel lymph node biopsy following neoadjuvant chemotherapy from 2018 to 2020. *SLNs* sentinel lymph nodes

**Fig. 4:**
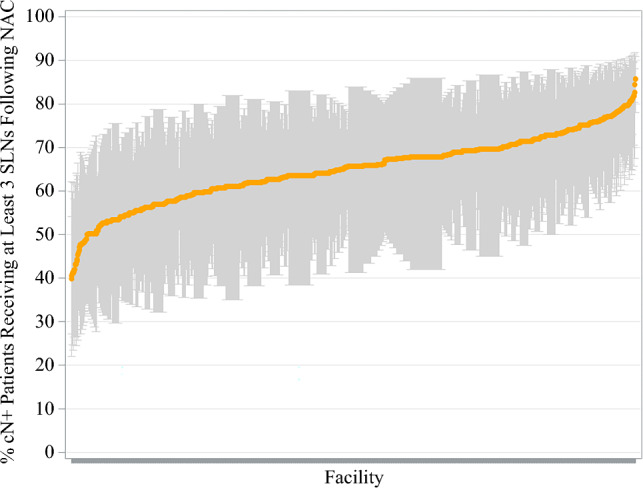
Facility-level variation in obtaining three or more nodes during SLN biopsy from 2018 to 2020. *SLN* sentinel lymph node, *NAC* neoadjuvant chemotherapy

Facility-level lymph node yield is summarized in Table 4. Patients undergoing SLNB following neoadjuvant chemotherapy from 2018 to 2020 had a mean of 4.1 (standard deviation [SD] 1.4) lymph nodes removed overall. Variation in the mean number of lymph nodes examined for SLNB ranged from 2.1 to 16.3. From 2018 to 2020, the rates of obtaining three or more SLNs remained the same during SLNB, at approximately 66% (Fig. 3). Although these rates remained relatively constant overall, there was significant interfacility variation in achieving this quality metric, ranging from 40 to 86%, with a median of 66% (IQR 61–70%) across facilities (Fig. 4). Differences in the rates of obtaining three or more SLNs were notable among facility type, with academic centers having significantly higher rates (*p *= 0.008) and comprehensive cancer centers having lower rates (*p *= 0.020) compared with the average rate of other facility types (electronic supplementary material [ESM] Fig. 1 and ESM Table 1).

**Table 4:** Lymph node yield for axillary surgery procedures

Procedure	Reliability-adjusted yield		
	Mean (SD)	Median (IQR)	Range
SLNB^a^	4.1 (1.4)	3.8 (3.3–4.6)	2.1–16.3
ALND^b^	13.3 (2.3)	13.2 (11.6–14.7)	7.4–24.3
SLNB with completion ALND^b^	11.9 (2.7)	11.8 (10.0–13.6)	5.7–21.6
Percentage of three or more SLNs examined during SLNB^a^	64.9 (7.7)	65.7 (60.5–69.7)	39.8–85.7

*SD* standard deviation, *IQR* interquartile range, *SLNB* sentinel lymph node biopsy, *ALND* axillary lymph node dissection, *SLNs* sentinel lymph nodes

^a^Lymph node yield obtained from 2018 to 2020

^b^Lymph node yield obtained from 2012 to 2020

## Discussion

This is the first study to examine trends in axillary surgery with subsequent analysis of facility-level variation in lymph node yield in patients with cN1-cN2 disease undergoing SLNB post-neoadjuvant chemotherapy for breast cancer. These results help to understand the use of axillary surgery nationally, and we report some important findings. First, we have demonstrated significant de-escalation of axillary surgery, with a > 50% reduction in ALNDs performed. Second, we have observed that although more facilities are performing SLNB in this patient population, the rate of obtaining three or more lymph nodes during SLNB has mostly stayed the same over time, suggesting that facilities not adhering to this technical recommendation may perform inadequate staging, leading to a possible performance gap and an opportunity for improvement.

This study demonstrated that the national rates of ALND in women with cN1–cN2 breast cancer following neoadjuvant chemotherapy have decreased substantially, with a corresponding increase in the rates of SLNB since trial data established its feasibility. This is consistent with prior studies examining patterns in axillary surgery post-neoadjuvant chemotherapy.^10,14–16^ The rate of axillary surgery de-escalation was relatively rapid, with an almost 50% reduction in ALNDs occurring only 6 years after the publication of the ACOSOG Z1071 trial. Previous research has shown lag times of up to 17 years before research findings are adopted into clinical practice.^27^ This rapid adoption suggests centers are willing to de-escalate axillary surgery for patients with a complete pathologic response to neoadjuvant chemotherapy. This is supported by additional studies following ACOSOG Z1071 showing no difference in axillary recurrence rate, disease-free survival, and overall survival on short-term follow-up in cN1 patients who achieved a pCR following neoadjuvant chemotherapy and who underwent SLNB versus ALND.^28,29^ Parallels in practice can be seen with the rapid de-escalation of completion lymph node dissection in favor of SLNB in melanoma following the results of the Multicenter Selective Lymphadenectomy Trial-II.^30–32^ This potentially indicates that surgeons and patients consider lymphedema a major concern and are eager to avoid completion lymph node dissection if oncologic outcomes are not compromised.

Although there have been higher rates of SLNB performed following neoadjuvant chemotherapy, our study demonstrates that the rates of obtaining three or more SLNs in centers performing this procedure have remained relatively constant over time at approximately two-thirds of facilities. There was no subsequent increase in adhering to the recommend lymph node yield during this procedure as expected. This suggests that approximately one-third of facilities may perform staging with a higher false negative rate than clinically accepted. A closer evaluation of prior landmark studies addressing the feasibility of SLNB following neoadjuvant chemotherapy suggests that achieving this metric may be difficult. The SENTINA trial found a median SLN yield of two nodes obtained. In contrast, the SN FNAC trial noted an average (mean) SLN yield of 2.7 in patients with cN+ disease following neoadjuvant chemotherapy.^1,4^ Furthermore, the ACOSOG Z1071 and SENTINA trials demonstrated a lymph node yield of three or more SLNs in only 56% and 34% of patients, respectively.^3,4^

Recent prospective and retrospective studies have had mixed results in achieving this technical consideration. A prospective study by Mamtani and colleagues demonstrated a median SLN yield of four, and retrieved three or more SLNs in 86% of its cohort with cN1 disease following neoadjuvant therapy.^33^ Similarly, in a retrospective study by Wong et al., a median SLN yield of four was attained in patients with cN1–cN2 disease who had a complete pathologic response to neoadjuvant chemotherapy.^11^ In contrast, following neoadjuvant therapy, the GANEA 2 study saw a median SLN yield of two in patients with cytologically proven axillary disease.^2^ A single-institution study in Italy reported a median lymph node yield of two in cN1-cN2 patients, and found that fewer than three SLNs were removed in 74% of patients.^9^ Although SLNB is a procedure that does not solely rely on visual inspection to identify the target node, substantial variability in yield still exists with the localization tools available. However, it is important to note that obtaining three SLNs is a surrogate marker for adherence, and it is unclear if failure to obtain three SLNs following neoadjuvant chemotherapy would have significant implications for patient outcomes. Data on axillary and/or regional recurrence, disease-free survival, and overall survival are needed on long-term follow-up studies, which are not available at this time.

Our results demonstrated a wide variation in SLN yield by facility. Variation in SLN yield can be due to factors such as obesity, tumor location other than the upper outer quadrant, tumor histology, patient age, and race.^34–36^ It is also known that variation exists in the pathologic evaluation of lymph nodes, which may contribute to observed differences.^37^ Although no work has specifically evaluated the effect of neoadjuvant chemotherapy on SLNB yield, prior studies have noted that lymph node yield in ALND following neoadjuvant chemotherapy is lower than expected. Explanations for this center on biological changes in the lymph nodes due to chemotherapy with a subsequent pCR. This can lead to eradication of the tumor in lymph nodes and replacement by scar, shrinkage of the tumor, or regression of lymphoid tissue, making evaluating the lymph node more difficult for the pathologist.^38–40^ This same process also likely occurs for all positive nodes affecting SLNB.

Targeted removal of the biopsy-positive clipped node has been shown to substantially lower the false negative rate to an acceptable level.^5^ The biopsy-positive clipped node is not always an SLN, which can likely result in variation in nodal yield, although there is more certainty that the appropriate node has been removed. It has recently been shown that patients undergoing targeted axillary dissection following neoadjuvant chemotherapy had an increased breast cancer-specific survival and lower locoregional recurrence rate compared with patients undergoing targeted axillary dissection with ALND on short-term follow-up.^41^ This highlights that the role of targeted axillary dissection in this patient population will likely increase in use.

The current variation in SLNB lymph node yield following neoadjuvant chemotherapy in patients with cN1–cN2 disease suggests a need for standardization. This represents an area of improvement and a possible opportunity for the Operative Standards in Cancer Surgery program to reduce variation and improve quality. The American College of Surgeons has established the operative standards in cancer surgery to detail critical elements of cancer surgery that should be performed to improve surgical technical quality.^42^ Currently, 134 operative standards have been established across 15 cancer types. Of those established, six standards have been implemented since 2020, of which breast cancer is included. The current standards in breast cancer surgery target the necessary steps to perform an adequate SLNB and ALND;^43^ however, special technical considerations in patients who have undergone neoadjuvant chemotherapy may improve lymph node yield during axillary surgery. The operative standards in cancer surgery video series were created to supplement the written format to aid surgeons in performing the optimal technique during critical portions of a procedure.^44^ Consideration of adding videos for SLNB performed in node-negative and node-positive axilla post-neoadjuvant chemotherapy to note differences in the operative field and technique could serve as a valuable tool for practicing surgeons treating breast cancer.

Important limitations should be noted as they relate to our study. First, we performed a retrospective analysis of the NCDB, which is vulnerable to documentation and classification errors of key variables such as the type of axillary surgery performed. However, the NCDB is the primary reporting method of the Commission on Cancer, and data are reported in a standardized fashion using certified tumor registrars who undergo training for cancer registries. Second, this study did not evaluate whether the biopsy-positive clipped node was removed during SLNB, a variable not yet available in the NCDB, and could have altered lymph node yield as retrieval of the clipped node is associated with a low, acceptable false negative rate regardless of the total number of nodes removed. However, the use of this technique to lower the false negative rate is only performed at select centers, which is likely only representative of some facilities in this study. Third, we have concluded that there has been minimal change in the retrieval rates of at least three lymph nodes during SLNB among facilities when our study only included 3 years of lymph node yield for this procedure. There could have been a more robust increase in prior years that was not captured in our study. However, by using the dedicated variable to the SLNB examination, which has only been available recently, we are more confident that the yields obtained accurately reflect the current facility-level variation.

## Conclusion

SLNB following neoadjuvant chemotherapy in patients with cN+ breast cancer with a pCR is feasible and surgical trends show significant de-escalation of axillary surgery with increasing use of SLNB alone in this clinical setting. Techniques to minimize the false negative rate of the procedure include the removal of three or more lymph nodes during SLNB. We have demonstrated that although de-escalation of axillary surgery has occurred with fewer patients undergoing ALND, there has overall been no significant change in the rates of obtaining three or more lymph nodes during SLNB following neoadjuvant chemotherapy. More importantly, there is substantial facility-level variation in achieving this metric, suggesting an opportunity for improvement to improve patient outcomes.

## Supplementary Information

Below is the link to the electronic supplementary material.

Supplementary file1 (DOCX 134 KB)
