# Supplementary material for: National Variation in Implementation of Sentinel Lymph Node Biopsy for Clinically Node-Positive Patients Undergoing Neoadjuvant Therapy
Source: Ann Surg Oncol. Author manuscript; Available in PMC 2025 Jul 1. (PMC12130160; doi:10.1245/s10434-025-17293-x)
Supplement: Supplementary File [file NIHMS2078595-supplement-Supplementary_File.docx]

**SUPPLEMENTARY MATERIAL**

**TABLE 1** Reliability-adjusted rates of obtaining three or more nodes during sentinel lymph node biopsy by facility type from 2018 to 2020

| Facility type | Mean (95% CI) | Median (IQR) | Range |
| --- | --- | --- | --- |
| Unknown | 65.6% (63.6–67.6%) | 67.8% (62.0–67.8%) | 53.4–79.6% |
| Community Cancer Center | 64.5% (63.7–65.3%) | 64.1% (61.1–69.3%) | 40.5–77.2% |
| Comprehensive Cancer Center | 63.7% (62.9–64.4%) | 63.9% (58.6–68.9%) | 41.1–81.7% |
| Academic Cancer Center | 67.2% (66.1–68.3%) | 68.2% (62.2–72.7%) | 41.9–84.5% |
| Integrated Network Center | 65.7% (64.8–66.6%) | 66.1% (61.3–70.9%) | 39.8–85.7% |

**FIG. 1** Facility-level variation in obtaining three or more nodes during sentinel lymph node biopsy by facility type from 2018 to 2020

| **Community Center**  **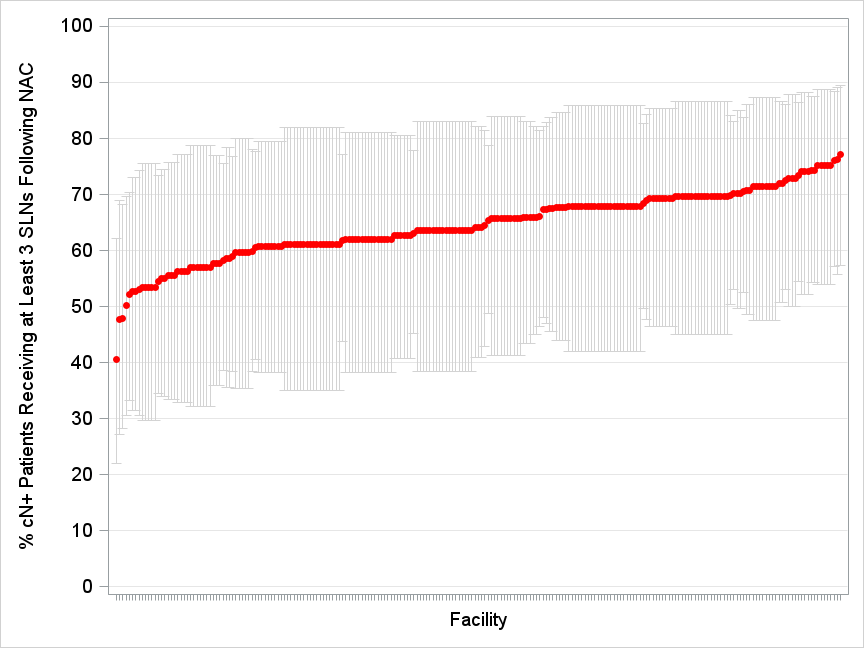** | **Comprehensive Center**  **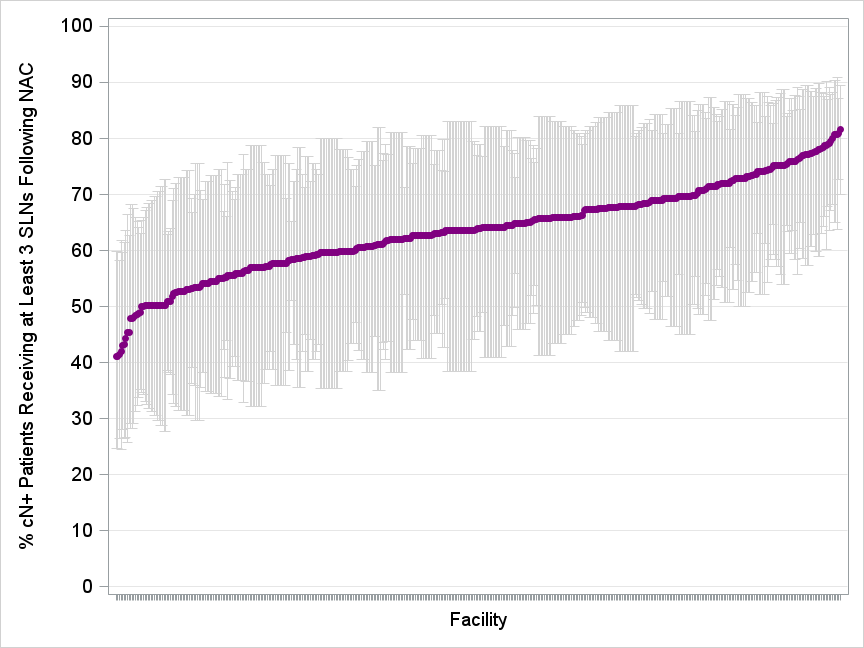** |
| --- | --- |
| **Academic Center**  **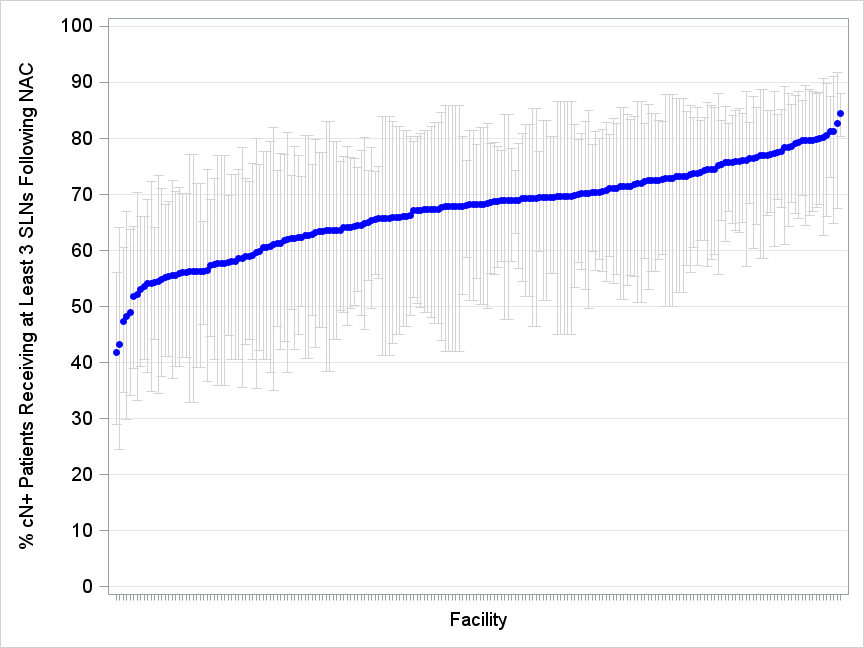** | **Integrated Network**  **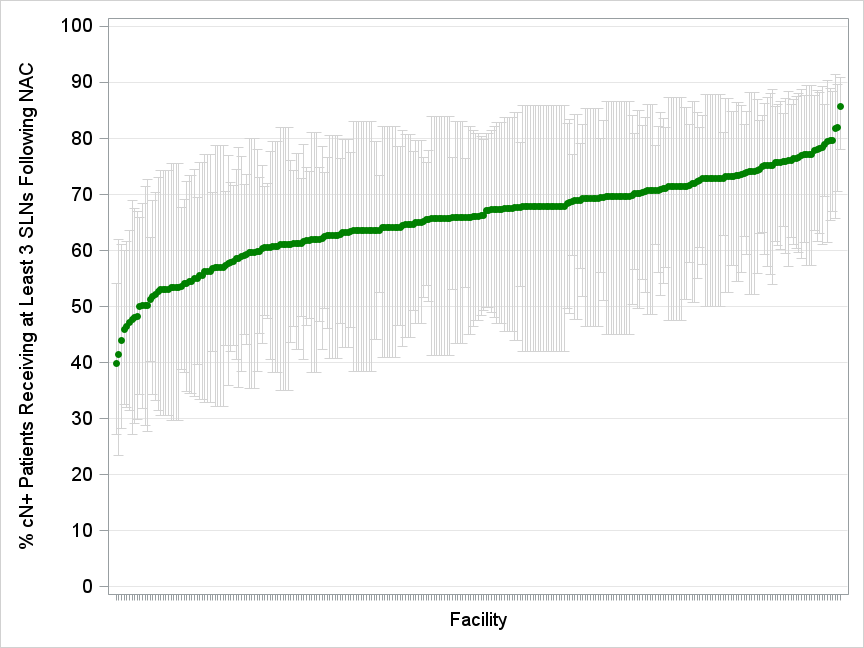** |
